# DNA methylation results depend on DNA integrity—role of post mortem interval

**DOI:** 10.3389/fgene.2015.00182

**Published:** 2015-05-18

**Authors:** Mathias Rhein, Lars Hagemeier, Michael Klintschar, Marc Muschler, Stefan Bleich, Helge Frieling

**Affiliations:** ^1^Laboratory for Molecular Neuroscience, Department of Psychiatry, Social Psychiatry, and Psychotherapy, Hannover Medical SchoolHannover, Germany; ^2^Department of Legal Medicine, Hannover Medical SchoolHannover, Germany

**Keywords:** DNA degradation, quality, epigenetic, post mortem

## Abstract

Major questions of neurological and psychiatric mechanisms involve the brain functions on a molecular level and cannot be easily addressed due to limitations in access to tissue samples. Post mortem studies are able to partly bridge the gap between brain tissue research retrieved from animal trials and the information derived from peripheral analysis (e.g., measurements in blood cells) in patients. Here, we wanted to know how fast DNA degradation is progressing under controlled conditions in order to define thresholds for tissue quality to be used in respective trials. Our focus was on the applicability of partly degraded samples for bisulfite sequencing and the determination of simple means to define cut-off values. After opening the brain cavity, we kept two consecutive pig skulls at ambient temperature (19–21°C) and removed cortex tissue up to a post mortem interval (PMI) of 120 h. We calculated the percentage of degradation on DNA gel electrophoresis of brain DNA to estimate quality and relate this estimation spectrum to the quality of human post mortem control samples. Functional DNA quality was investigated by bisulfite sequencing of two functionally relevant genes for either the serotonin receptor 5 (SLC6A4) or aldehyde dehydrogenase 2 (*ALDH2*). Testing our approach in a heterogeneous collective of human blood and brain samples, we demonstrate integrity of measurement quality below the threshold of 72 h PMI. While sequencing technically worked for all timepoints irrespective of conceivable DNA degradation, there is a good correlation between variance of methylation to degradation levels documented in the gel (*R*^2^ = 0.4311, *p* = 0.0392) for advancing post mortem intervals (PMI). This otherwise elusive phenomenon is an important prerequisite for the interpretation and evaluation of samples prior to in-depth processing via an affordable and easy assay to estimate identical sample quality and thereby comparable methylation measurements.

## Introduction

Investigating the brain and its molecular regulation is limited to either translational animal models or post mortem investigations. Both approaches have their specific advantages and limitations. Animal models reduce inter-individual variance and enable a strict observation of experimental and environmental conditions, including the sacrifice of the animal. However, the translational value of certain pathologies, especially in the field of psychiatric molecular research, is very limited. With post mortem studies the possibilities for anamnesis are only limited by differences in the evaluation of certain phenotypes by different psychiatrists, but all life parameters such as age, drug treatment, agonal state and cause of death are highly variable in comparison. Another important caveat to consider is that, depending on the type and progression of causality leading to death, the parameters observed in the study might be seriously deregulated, since it is widely accepted that epigenetic regulation, especially in the context of drastic metabolic impacts, is altered in the range of minutes to hours (McCullumsmith and Meador-Woodruff, 2011).

After the publication of epigenetic preservation of methylation patterns in archeological subjects (Llamas et al., 2012) the preservability of epigenetic readings seemed secured, if the DNA was amplifiable. DNA stability in Post mortem tissue has been investigated extensively already (Bär et al., 1988; El-Harouny et al., 2009). Studies by Barrachina and Ferrer et al. describe the preserved epigenetic analysis quality of brain tissue up to a PMI of 48 h (simulated) for two genes (Barrachina and Ferrer, 2009) as well as the general ambiguity involved with the already mentioned interindividuality of samples in respect to protein, RNA and DNA degradation (Ferrer et al., 2008). Large-scale epigenetic studies are also performed in human post mortem brain tissue as well as comparative studies to investigate similarities and differences between blood and brain (Rollins et al., 2010; Wockner et al., 2014). While these studies provide information about DNA quality they not always refer to respective PMI timepoints. We were curious whether there might be differences in the determination of methylation results that limit such approaches. For the collection of new post mortem collectives it would also be beneficial to have a fast assay that provides first evidence for the analysis of reliable data while extending the PMI window as far as possible.

We therefore decided to perform a longitudinal pilot study where blood and brain tissue was kept at ambient temperatures and frozen at distinct timepoints. We observed a correlation for the progression of DNA degradation both on the level of macroscopic DNA and on the readouts of the epigenetic analysis from typical target genes in the field of psychiatry and neurology, the serotonin transporter gene (*SLC6A4*) promoter (Philibert et al., 2008; Vijayendran et al., 2012; Abdolmaleky et al., 2014) or aldehyde dehydrogenase 2 (*ALDH2*) (Ikawa et al., 1983; Kimura et al., 2009; Xue et al., 2012). To reaffirm the quality estimation method established here we compared the pig brain data with actual samples from an ongoing post mortem trial with known post mortem intervals. We thereby report a first indication for the possible impact DNA stability has on the analysis of epigenetic phenomena in post mortem cohorts.

## Materials and methods

### Collection and processing of porcine samples

**Figure 1:**
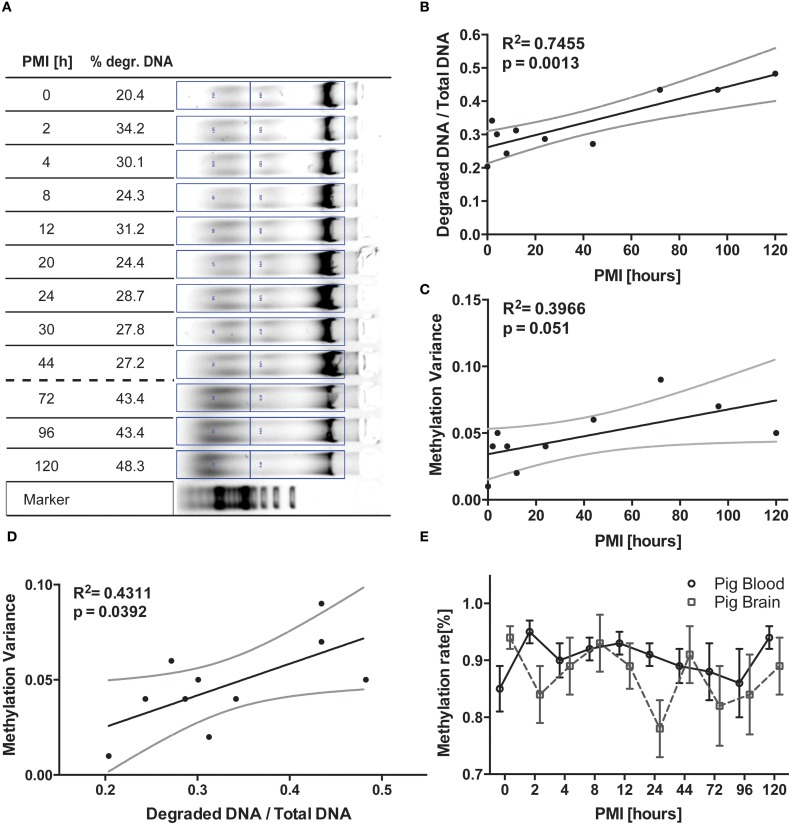
**Longitudinal pig trial. (A)** DNA was isolated from pig brain tissue frozen at timepoints depicted and separated on a 0.7% agarose gel. Degraded DNA was put in relation to total DNA in the respective lane for normalization. **(B)** The respective graphical display of the increasing degradation ratio (degraded DNA/total DNA) and **(C)** likewise increasing variance of methylation data plotted against PMI were analyzed via linear regression. Variance was calculated from methylation analysis of brain cortex DNA (*n* = 2, means of 8 CpGs in the regions analyzed). These two resulting values are then correlated in **(D)** via linear regression, demonstrating a positive correlation between relative amount of degraded DNA and variance of methylation. **(E)** Finally, mean methylation is plotted against respective PMI for the two tissues analyzed. All black lines represent the linear function the statistics are based upon while grey lines represent 95% CI. The error bars in **(E)** are ±SEM.

Heads as well as blood from sacrificed pigs (see Table 1) were immediately retrieved from the Department of Cardiothoracic, Transplantation and Vascular Surgery, Hannover Medical School, Hannover, Germany. The head was removed for transport on ice within 30 min after death. The animals included were not older than 4 months to avoid bias through age- related DNA damage. Within 1 h post mortem, the skullcap was opened to allow access to the brain for sample asservation. The lid of the skull was retained to be able to close the head again and thereby keep the influence of the opening as minimal as possible. The head was stored at 19–21°C room temperature. All the samples were extracted from the Frontal gyri of the porcine cerebrum with a sharp scalpel. The weight varied between 20 and 50 mg while cutting depth was less than 1 cm, thereby ensuring not to extract other regions than cerebrum and maintain comparability of samples. Time Point 0 depicts the time 1 h after the actual removal of the head, when the first sample of pig cortex was frozen at −80°C together with blood aliquots. At 0, 2, 4, 8, 12, 24, 44, 72, 96, and 120 h post mortem cortex samples were frozen together with heparin-treated blood aliquots prepared within the first hour post mortem. From timepoint 6 (24 h) on the removal of samples was ±2 h. The first pig included more timepoints for measurement (20, 30, see Figure 1A), which were not included for the second pig. We decided not to cut out the respective columns from picture 1A but removed them from further analyses in order to merge methylation data points with the second animal.

**Table 1:** **Age and weight of the pigs used for the longitudinal investigation**.

**Animal**	**Age (months)**	**Weight (kg)**
P1	2, 5	22, 6
P2	4, 5	61

### Collection and processing of human samples

Human samples were collected at the Department of Legal Medicine, Hannover Medical School. The local ethics committee approved the collection of samples that were collected during the standard mandatory autopsy performed to exclude homicide and clarify the cause of death (approval #1127-2011, Hannover Medical School, Hannover, Germany). The six brain regions collected were: nucleus arcuatus, nucleus accumbens, corpus amygdalum, corpus mamillarium, ventral tegmental area, and the dorsal hippocampus. Subject age, lifestyle, cause of death and PMI vary significantly (see Table 2).

**Table 2:** **Biometrical data for the human collective that was investigated**.

**Patient#**	**Alcohol abuse**	**Sex**	**Age**	**Treatment**	**PMI**	**Smoker**
2	No	Male	45	None	40	No
3	Yes	Male	47	Chlorproxithem, Oxazepam	68	Yes
5	Yes	Female	56	Blood pressure, analgetics	52	N.A.
11	No	Female	60	Intensive care	47	N.A.
12	No	Male	63	Blood pressure	38	N.A.
13	Yes	Male	57	N.A.	52	Yes

### DNA isolation and bisulfite reaction

DNA was purified through standard techniques [Trizol, VWR, Radnor, USA; automated DNA cleanup using Nucleomag bead kit from Macherey&Nagel, Düren, Germany on a Biomek NxP pipetting robot (Beckman Coulter, Brea, USA)]. Bisulfite conversion of DNA samples then were bisulfite-converted and purified by the EpiTect® 96 Bisulfite Kit (QIAGEN AG, Hilden, Germany) according to the manufacturer's recommendations.

### DNA gel preparation

After determining DNA concentration using a Nanodrop (Peqlab/ VWR, Radnor, USA) 250 ng DNA from each sample was loaded onto a 0.7% agarose gel containing 0.004% ethidium bromide (AppliChem, Gatersleben, Germany) together with 3 μl 100 kb DNA ladder (VWR, Radnor, USA) and separated at 100 V constant for 45 min at room temperature and a picture taken with a Geldoc XR+ molecular imaging system (Bio-Rad Laboratories, Hercules, USA). Relative quantification was calculated dividing equal area values of genomic DNA degradation smear with the respective total amount of DNA in the lane, resulting in a ratio of % degenerated DNA compared to total DNA that could then be compared across samples and time points (ImageLab software 5.2; Bio-Rad Laboratories, Hercules, USA) (see Figure 1A, upper/lower region of the respective lanes).

### Bisulfite conversion of DNA, PCR strategy and sequencing

Bisulfite sequence primers were designed using the software packages MethylPrimer Express (ABI Life Technologies, Grand Island, USA), Geneious (Biomatters, Auckland, New Zealand) and the Netprimer tool (PREMIER Biosoft International, Palo Alto, USA) for validation of different melting temperatures. Primers were ordered from Metabion (Steinkirchen, Germany). Touchdown PCRs (Korbie and Mattick, 2008) with starting temperatures of 70°C (*ALDH2*) and 62°C (*SLC6A4*), respectively, were performed using a semi-nested strategy and sequenced on a 3500XL genetic analyzer from ABI Life Technologies (Grand Island, USA) (see Table 3 for primer details).

**Table 3:** **Primers used for the Touchdown PCRs**.

**Primer name**	**Sequence**	**Fragment size**
ALDH2-F2_NEU	GAGGTATGGTTGTGTGATTG	258 bp
ALDH2-F1_NEU	TTTGGTGTTGAAATTAGAGTT	
ALDH2_RC1_NEU	ACTCACTACAAACTCTACCTCC	
5-HTTp-T1_1F_208	GAAATGAAGTTAGTTGGAAGGT	378 bp
5-HTTp-T1_2F_210	GGGTTTAGATTTTTATTTTATGAT	
5-HTTp-T1_2RC_211	AACAACCTAACTATTCCCAACT	
5-HTTp-T1_1RC_209	CCCTCTACGAAACACCCTTA	

Analysis of CpG-specific methylation percentage was processed by the Epigenetic Sequencing Methylation (ESME) analysis software (Lewin et al., 2004) and the methylation rate (%) of each CpG site within the amplified region was estimated by the ratio between normalized peak values of Cytosine (C) and Thymine (T).

### Statistical analysis

All statistical calculations were performed using IBM Software Package for the Social Sciences (SPSS, IBM, Armonk, USA) or Prism 6 (Graphpad, LaJolla, USA). Residuals were assessed for normality within each group using the Shapiro-Wilk (W) goodness-of-fit test. Methylation measurements deviated significantly from normality, shifting the analysis to non-parametric tests. Positions with less than 70% coverage were excluded from analysis.

Calculation of variance follows the formula var = mean[(*x* – mean(*x*))^∧^2]. All values for variance were calculated in SPSS.

Residuals for both degradation [*W*_(10)_ = 0.531, *p* = 0.009] and variance [*W*_(10)_ = 0.163, *p* = 0.015] of pig brain samples did not deviate from normality, in consequence we applied the Pearson correlation test. Linear regression was then performed without permutation of values, since we expected a direct interaction between both parameters.

Human methylation data was tested for significant differences between pooled brain measurements for each patient via the Student's *t*-test and found equal according to Tukeys correction for multiple comparisons.

## Results

### DNA degradation series in pig blood and brain

In the porcine brain samples we were able to detect the progressing degradation of DNA by standard gel electrophoresis and semi-quantification of incremental increase of sheared DNA in the sample (Figures 1A,B). This degradation curve was not identically reproducible in the blood samples as well as not reproducible in the second pig analyzed due to interindividual differences in the degradation kinetics (data not shown), although time-dependent progression was visible (Compare to Figure S1).

### Methylation analysis of pig blood and brain samples

For the two animals investigated, in addition to the progressing macroscopical changes observed during gel electrophoresis, we noted an increase in variance of mean methylation percentage with advancing timepoints (See Figure 1C, *R*^2^ = 0.3966, *p* = 0.051). Pearson correlation of the parametric variables showed robust significance between the two parameters (*F* = 9.595, *R*^2^ = 0.545, *p* = 0.0147) the mean variance of brain measurements of the two subjects analyzed plotted against the degradation values calculated from the DNA gel shows a good correlation (Figure 1D, *R*^2^ = 0.431, *p* = 0.0392) of data points. We also tested blood samples for the according comparison, but while the DNA gel picture showed similar degradation patterns, the gel was not quantifiable in the way the brain samples have been (data not shown). This is supposedly due to the artificial storage of the blood aliquots in vials rather than in the corpse, thereby altering the natural exposure to degradation processes. However, upon calculating the variance for the epigenetic results, we were able to equally correlate these values to the progress of degradation observed in brain tissue (Figure S1, *F* = 5.422, *p* = 0.0483, *R*^2^ = 0.4040).

Across all samples generated at all timepoints, the methylation rate measurements did not vary significantly from each other (Figure 1E, see also Figure S3). Some of the CpG positions were harder to sequence, but this deviation is consistent across all the timepoints pointing toward unknown interindividual differences (data not shown). Since single CpG positions were equally affected by the degradation phenomenon, we decided to limit our analysis to the change observed in the whole region (see Figure S2).

### DNA degradation test in human brain regions and blood

**Figure 2:**
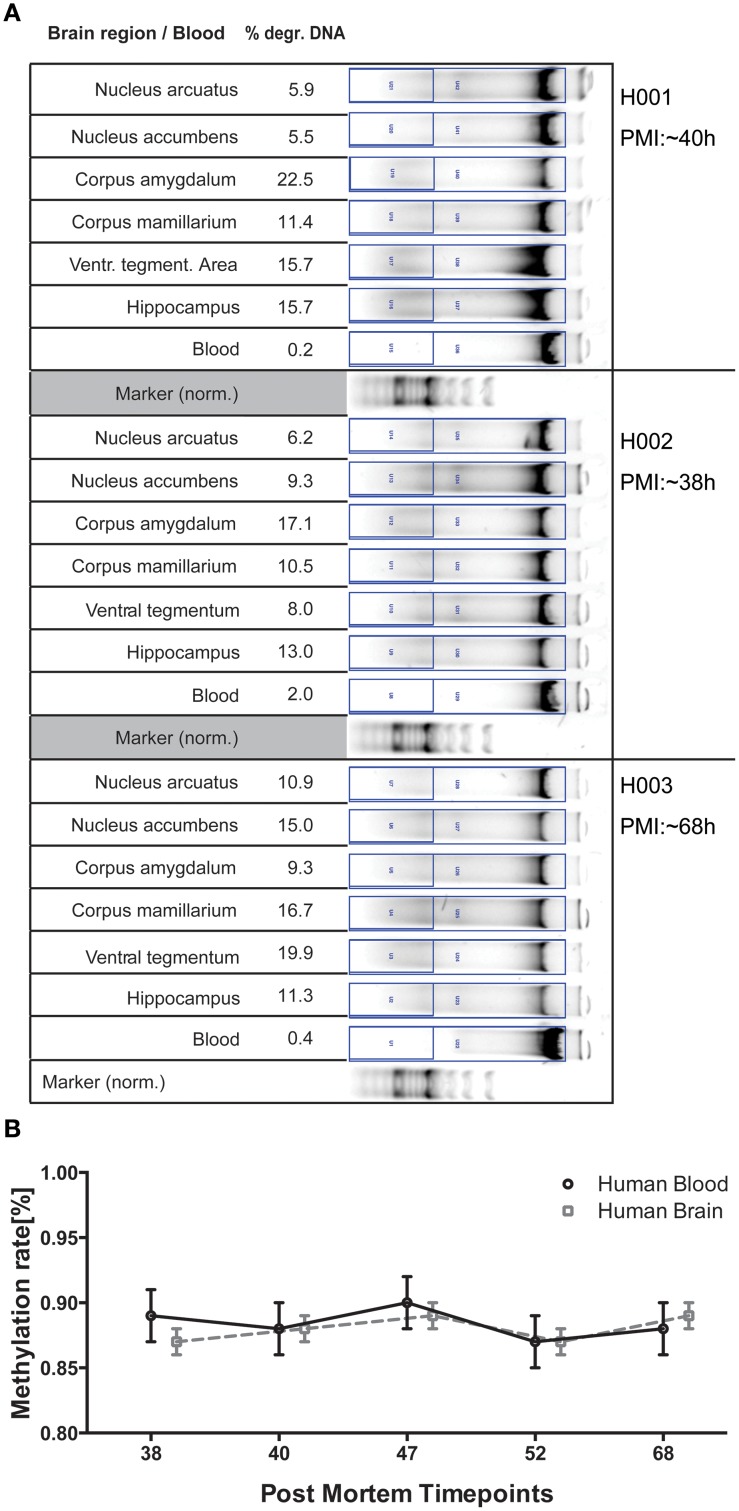
**(A)** DNA gel electrophoresis for 3 of the six human subjects analyzed for comparison. Degraded DNA was put in relation to total DNA in the respective lane for normalization. **(B)** Epigenetic variance of the ALDH2 gene for timepoints investigated in human DNA is stable both between blood and brain as well as in regards to variance of measurements. The mean methylation rate (%) of 10 CpGs of 6 brain regions was calculated for 5 post mortem subjects and is plotted against respective PMI for the two tissues analyzed. All error bars are ±SEM.

In an ongoing post mortem trial we are collecting blood and brain samples from 6 different regions. These subjects vary strongly in all parameters assessed (see Table 2 in Materials and Methods) and were selected for covering the range of PMIs collected until now (38–68 h). DNA degradation displayed a much more diverse picture compared to the pig trial data, which reflects the highly individual gender, origin, lifestyle, pathologies, cause of death, and environmental circumstances at the site of death (see Figure 2A). However, epigenetic measurements show only small deviations from the methylation rate (%) for both blood and the different brain regions (not significant), proving the integrity of samples collected within this PMI range (Figure 2B).

## Discussion

DNA is an amazingly resistant molecule. From forensics to archeology this feature is the key factor necessary to recollect information from degraded tissue of any kind, utilizing the information conserved in this resilient molecule (Wockner et al., 2014). Most astoundingly, even in selected cases of archeological samples methylation analysis is possible if the material can be amplified (Llamas et al., 2012). In this study we wanted to determine the validity of such investigations in a longitudinal setup to ensure the precision of data recovered is also true for highly variable regions of methylation. While the influence of PMI on epigenetic measurements has already been investigated (Barrachina and Ferrer, 2009), we were curious to see whether there will be an observable influence when the PMI is increased to the point where a safe extraction of individual regions is no longer possible due to tissue degradation. Our first observation was actually backing up the findings of previous authors in terms of consistency, as we were not able to detect any significant time-dependent effect of the mean epigenetic readouts themselves. Noting a visible increase in the deviation error bars, we decided to test for change in variance. With differences between cohorts seldom yielding results above 5–20% methylation, an increase in sample deviation could potentially lead to false interpretation of observed effects. We therefore calculated the mean variance for the brain analysis from the two subjects and plotted the resulting data against the normalized degradation values from the gel. Most interestingly these values correlate significantly (*p* = 0.0147) and provide a statistical explanation for 43% of cases (Figure 1D). Therefore, the influence of PMI for the timeframe of investigation not only proves the connection of measurement quality with the amount of degradation but also that PMI and methylation variance play an essential role in the fidelity of data measured. Since both degradation and methylation variance increased with timepoint 8 (72 h) we decided to take the respective calculated degradation ratio as a threshold for our sample collection in the ongoing post mortem trial. Also our suggested degradation ratio seems to suggest a ratio of 40% as a tentative threshold for DNA quality. However, taking the variability of the results for different tissues into account, we need to prove this assumption in further experiments.

To see whether our DNA quality prediction holds true, we analyzed 6 human subjects for blood and brain degradation in perspective of their estimated PMIs. Since only samples from timepoints below 72 h PMI were collected, we do not have a comparable PMI included for the other timepoints generated in the pig trial. Region-specific differences of DNA degradation are expected to be a multifactorial phenomenon influenced not only by PMI, but also lifestyle influences, cause of death and environmental conditions. The samples therefore reveal individual patterns concerning degradation or methylation variance. In spite of these factors both blood and brain sample methylation varied only in small degrees from the global mean in both value and variance, reaffirming overall tissue quality to still provide reliable data. We also observed small tissue-specific methylation differences for *ALDH2* between blood and brain, pointing toward the translational capacities for investigation of this locus in larger patient studies for a potential prognostic value in diagnosis and therapy (Figure 2B).

There are a couple of limitations to this study that narrow the scope of interpretation of these findings. The number of included subject, counting two for the pig experiment and 6 for the human post mortem cohort needs replication as sample size only allows for preliminary interpretations. It is remarkable though that even for these small cohort sizes, methylation analysis displays only small interindividual variation and a significant trend in methylation variance in the pig brain samples (Figure 1C). The limitation to cortex tissue in the pig trial is partly compensated statistically by the analysis of six regions in the human subjects, creating technical duplicates. A specific degradation resulting from unavoidable continued exposure to air during the different PMI timepoints of the pig trial is also introducing trial-specific artifacts. Observing the general effect variance dependence on PMI, we are planning to follow up this pilot study with an investigation of this phenomenon in a genome-spanning setting. While we believe to observe a general phenomenon based on investigation of genuinely different tissues and two genes involved in different metabolic processes (alcohol metabolism and neuronal signal transmission), as of now it cannot be ruled out that the effect is limited to these specific loci.

This holds true for blood methylation levels as well when related to the brain degradation values calculated for the respective timepoints. Analysis of epigenetic variance in pig blood suggests equal correlations in terms of time-related decrease in quality (Figure S1). While the effect of extracorporal storage of heparinized blood is to be taken into account, comparing the degradation patterns for blood and brain in human samples (Figure 2A) suggests that tissue-specific degradation thresholds need to be determined for gel electrophoresis. However, observing the strong variation of DNA quality in different tissues, the measurement in one specific region cannot be extrapolated on the whole subject, thereby potentially complicating DNA quality assessment.

Since the effect of degradation should be visible throughout the different tissues and genes and the human data is only a confirmatory test, we decided to take the available human ALDH2 gene information for comparison. We also had a good indication that variance for ALDH2 methylation is minimal in freshly obtained blood samples from previous human studies. We nevertheless cannot rule out the possible predisposed difference in variance between genes to influence the data displayed.

We hereby describe for the first time that epigenetic measurements are potentially influenced by PMI. In the process though, with DNA degradation we also discovered a simple way to ensure integrity of DNA quality from post mortem tissue that is able to predict variance of epigenetic data and therefore limit misleading interpretation of data generated by this technique. It is most likely that this finding impacts other techniques for measuring epigenetic phenomena, including next generation sequencing approaches. The impact of such a quality regimen is enormous, given that epigenetic differences discriminating conditions can very likely be in the range of 5–15% methylation difference. Further experiments will be needed to ensure the overall robustness of this method, but we are confident that elimination inconsistencies in epigenetic analyses will be highly improved by applying the findings presented here.

### Conflict of interest statement

The authors declare that the research was conducted in the absence of any commercial or financial relationships that could be construed as a potential conflict of interest.

## Supplementary material

Figure S1 **Pig blood methylation variance is plotted against the degradation values derived from the semiquantitative degradation scale in pig brain**. Irrespective of interindividual tissue degradation patterns methylation variance in blood samples leads to strong correlation (*R*^2^ = 0.596, *p* = 0.0089). The dashed line represents 95% CI.

Figure S2 **Epigenetic measurements split up for tissue and individual animals on the 10 timepoints that were identical for both subjects**. This data is merged in Figure 1E. P1 and P2 describe the individual pigs investigated for the individual timepoints. Error bars (±SEM) resulted from merging sequencing data of the 8 CpGs in the promoter stretch −1396 to −1016 in relation to the transcription start site of porcine SLC6A4 (Ensembl).

Figure S3 **Display of single CpG methylation pattern for Blood (A) and Brain (B) of the SLC6A4 gene promoter in the pig trial study**. Not seeing any meaningful difference between CpG methylation variance, we decided to compare global values instead of individual alterations. CpG 4 was excluded from this display as well as from analysis due to bad sequencing quality.
