# Supplementary figures and images for: DNA methylation results depend on DNA integrity—role of post mortem interval
Source: Front Genet. 2015 May 18;6:182. doi: 10.3389/fgene.2015.00182 (PMC4435253; doi:10.3389/fgene.2015.00182)

**Figure S1**

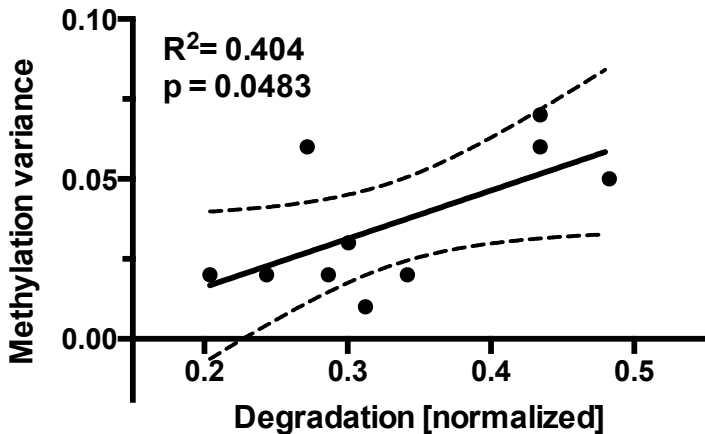

Supplement: Figure S1 — Pig blood methylation variance is plotted against the degradation values derived from the semiquantitative degradation scale in pig brain. Irrespective of interindividual tissue degradation patterns methylation variance in blood samples leads to strong correlation (R2 = 0.596, p = 0.0089). The dashed line represents 95% CI. [file Image1.PDF]

Figure S2

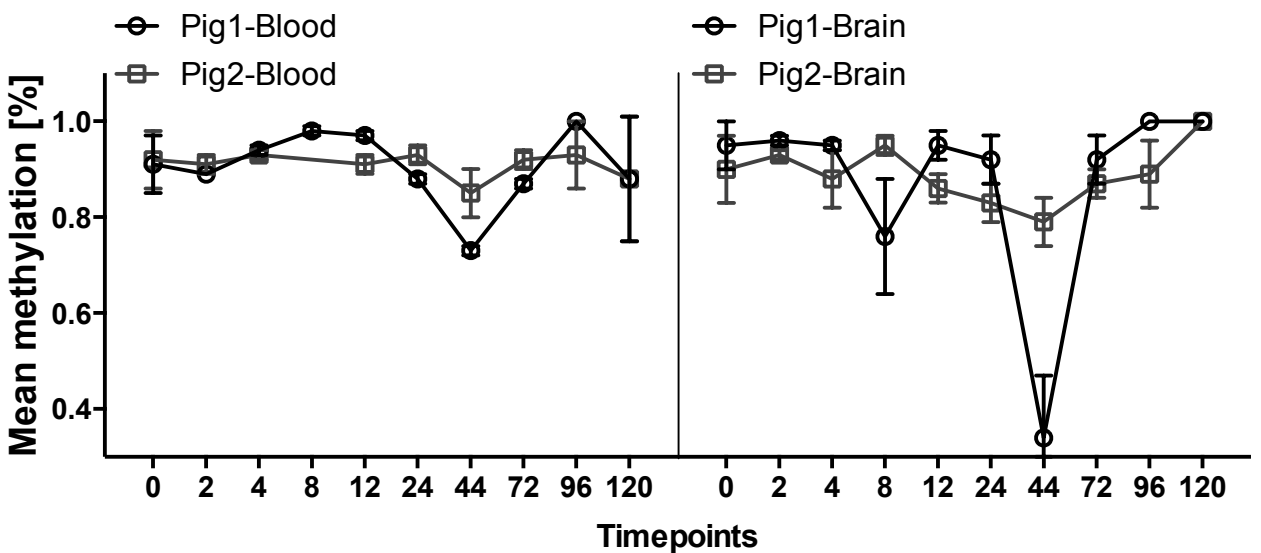

Supplement: Figure S2 — Epigenetic measurements split up for tissue and individual animals on the 10 timepoints that were identical for both subjects. This data is merged in Figure 1E. P1 and P2 describe the individual pigs investigated for the individual timepoints. Error bars (±SEM) resulted from merging sequencing data of the 8 CpGs in the promoter stretch −1396 to −1016 in relation to the transcription start site of porcine SLC6A4 (Ensembl). [file Image2.PDF]

Figure S3

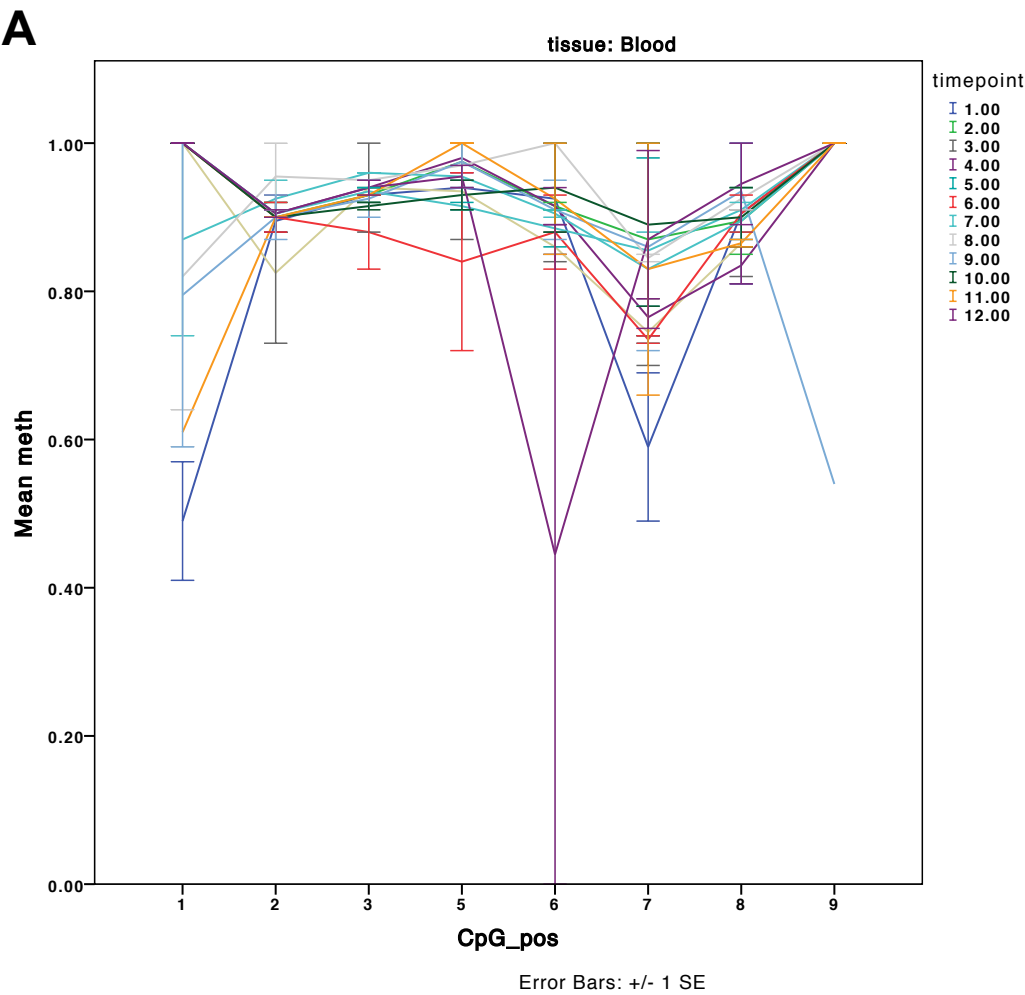

**B**

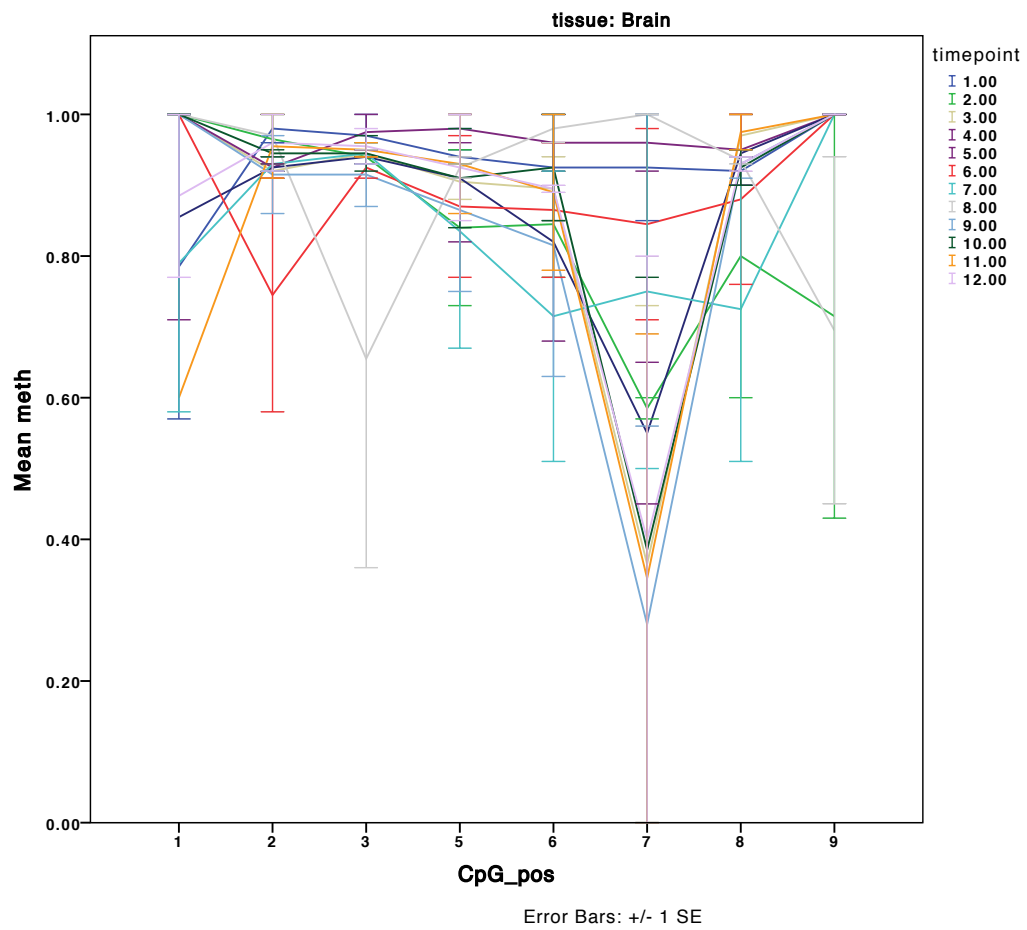

Supplement: Figure S3 — Display of single CpG methylation pattern for Blood (A) and Brain (B) of the SLC6A4 gene promoter in the pig trial study. Not seeing any meaningful difference between CpG methylation variance, we decided to compare global values instead of individual alterations. CpG 4 was excluded from this display as well as from analysis due to bad sequencing quality. [file Image3.PDF]
